# Weight loss with real-world doravirine use in the OPERA cohort: a US-based cohort study

**DOI:** 10.1186/s12981-025-00761-5

**Published:** 2025-06-21

**Authors:** Karam Mounzer, Laurence Brunet, Michael Sension, Ricky K. Hsu, Michael D. Osterman, Jennifer S. Fusco, Yohance O. Whiteside, Gregory P. Fusco

**Affiliations:** 1 Philadelphia FIGHT Community Health Centers, Philadelphia, LA USA; 2 Epividian, Inc, 150 Fayetteville Street Suite 2300, Raleigh, NC 27601 USA; 3 CAN Community Health, Fort Lauderdale, FL USA; 4 AIDS Healthcare Foundation and NYU Langone Medical Center, New York City, NY USA; 5 Merck & Co., Inc, Rahway, NJ USA

**Keywords:** HIV, Doravirine, Weight, Durability, Cohort, Real-world evidence

## Abstract

**Background:**

Weight gain has been associated with the use of antiretrovirals in people with HIV, especially with integrase inhibitors or tenofovir alafenamide, and among women. In 2018, doravirine became the latest non-nucleoside reverse transcriptase inhibitor to be approved in the US. We assessed changes in weight over time among virologically suppressed individuals who switched to a regimen containing doravirine (DOR).

**Methods:**

From the US-based OPERA cohort, treatment-experienced adults with HIV who switched to a DOR-containing regimen between 30AUG2018-30NOV2022 with a viral load < 50 copies/mL were included (followed through 31MAY2023). The study population was characterized and a linear mixed model was used to estimate rates of weight change on DOR. Results were stratified by sex, by patterns of efavirenz (EFV) and/or tenofovir disoproxil fumarate (TDF) use before/after switch to DOR, and by integrase inhibitor (INSTI) & tenofovir alafenamide (TAF) use combination (restricted to individuals who maintained the same combination before/after switch).

**Results:**

Of 388 included individuals, 21% were women, 33% were Black, and 78% were obese or overweight at DOR switch. Overall, people who switched to DOR lost an average of 0.80 kg/year (95% CI: -1.32, -0.28). Both women and men experienced statistically significant weight loss; women (70% Black, 70% aged ≥ 40 years) lost weight at a rate of -1.67 kg/year (95% CI: -3.32, -0.02) and men at a rate of -0.60 kg/year (95% CI: -1.12, -0.08). When EFV and TDF were absent before and after switch to DOR, statistically significant weight loss was observed. Among those who had the same INSTI and TAF combination throughout and had any INSTI or TAF use, a statistically non-significant trend toward weight loss was observed.

**Conclusions:**

In one of the first real-world analyses of weight changes among virologically suppressed individuals who switched to a DOR-containing regimen in the US, DOR was associated with statistically significant weight loss. Patterns of use of other antiretrovirals did not fully explain the observed weight loss. These findings are clinically meaningful given that most individuals included were overweight or obese at switch to DOR and that women were predominantly of perimenopausal or menopausal age.

## Background

In 2020, over 37 million people worldwide were living with HIV and 680,000 people died from AIDS [1]. Weight gain associated with ART use has been the focus of many studies, with weight gain linked to integrase strand transfer inhibitor (INSTI) and/or tenofovir alafenamide (TAF) use [2]. As for efavirenz (EFV) and tenofovir disoproxil fumarate (TDF), their use has been associated with weight loss [2], while their discontinuation has been associated with weight gain [3, 4]. Some weight gain has been reported with other antiretroviral (ARV) classes, including protease inhibitors and, to a lesser extent, non-nucleoside reverse transcriptase inhibitors (NNRTIs) [5]. Weight gain after initiation of certain ART regimens has also been demonstrated in women, especially in Black and Hispanic women [5–8], as well as in women of perimenopausal and menopausal age [9–11].

Doravirine (DOR) is a third generation non-nucleoside reverse transcriptase inhibitor (NNRTI) [12]. It was approved by the Food and Drug Administration (FDA) on 30AUG2018 as a single agent or co-formulated with lamivudine (3TC) and TDF as a single tablet regimen. DOR is dosed once daily and can be taken with or without food [13]. The efficacy of DOR has been established in clinical trials [14–16]. DOR-containing regimens also have an advantageous safety profile and have been tolerated as well as other agents in comparison studies [12, 14, 16].

Since the approval of DOR, little has been published about its real-world use in the US. As the latest NNRTI approved to treat adults with HIV, the role that DOR plays in the antiretroviral landscape is in constant evolution [17]. It is thus important to characterize the potential effect of DOR on clinical outcomes, including weight change, since its recent introduction to the US market. Assessing the impact of DOR in a large cohort representative of people with HIV in care in the US is the crucial first step towards understanding its role in the treatment landscape and assist in identification of individuals who could benefit from a DOR-containing regimen. Given the increasing concern of weight gain after initiation of ART, evaluating the potential effects of DOR on weight is particularly relevant in the current ARV landscape. This study aimed to characterize the real-world use of DOR and describe changes in weight over time among people with HIV who switched to a DOR-containing regimen.

## Methods

### Study population

This study was an observational cohort study of prospectively collected electronic health record (EHR) data from the Observational Pharmaco-Epidemiology Research and Analysis (OPERA^®^) cohort. At the time of this study, OPERA included data from 101 clinics in 23 US states and territories. People in OPERA represented approximately 15% of all people with HIV linked to care in the US [18]. OPERA complies with all HIPAA and HITECH requirements and has received annual institutional review board (IRB) approval by Advarra IRB, including a waiver of informed consent and authorization for use of protected health information.

All ART-experienced adults with HIV-1 who had their first DOR exposure between 30AUG2018 and 31DEC2022, were active in care (i.e., ≥ 1 visit within 24 months before/at time of switch to DOR), were virologically suppressed (viral load < 50 copies/mL), and had ≥ 1 baseline weight measurements (within 6 months before/at time of switch to DOR) and ≥ 1 follow-up weight measurement were included. Baseline was defined as the date of switch to the first DOR-containing regimen during the study period. Individuals were followed from baseline until the first of (a) discontinuation of DOR, (b) > 45 days without ART, (c) 12 months after last clinical contact, (d) death, or (e) study end (31MAY2023).

### Measurements

Baseline demographic and clinical characteristics were obtained from EHRs using the most recent entry at the time of or up to 6 months before switching to a DOR-containing regimen. Other ARVs taken concurrently with DOR in the baseline regimen and ARVs in the regimen directly prior to initiation of DOR were characterized. The primary outcome of interest was body weight (kg) at baseline and throughout follow-up.

### Statistical analyses

Descriptive analyses were conducted for all baseline characteristics and for treatment patterns, overall and stratified by sex. Absolute numbers and proportions were provided for categorical variables; medians and interquartile ranges (IQRs) were provided for continuous variables.

A linear mixed model with random intercept was used to assess average changes in weight over time on DOR while accounting for repeated measures. All weight measurements from baseline to the end of follow-up were used. A random intercept was included to account for differences in weight at time of switch. Time was modeled flexibly with restricted cubic splines. Knot placement was selected based on data distribution (0, 6, 12, and 24 months). An interaction term between time and stratification variables was included to assess changes in weight in all sub-groups of interest. Changes in body weight over time were estimated overall and stratified by sex.

### Sensitivity analyses

Two sensitivity analyses were conducted. The first controlled for the use of EFV and/or TDF, which have both been associated with weight loss [2–4]. Analyses described above for the main analysis were repeated, stratified by patterns of EFV and TDF use before and after switch to DOR: (a) TDF and/or EFV before & TDF after, (b) no TDF or EFV before & after, (c) TDF and/or EFV before only, and (d) TDF after only. This analysis was restricted to individuals for whom TDF and EFV use could be determined with certainty, excluding individuals with both DOR and EFV or both TDF and TAF recorded concurrently.

The second sensitivity analysis controlled for the impact of INSTI and/or TAF use, both of which have been associated with weight gain [2]. The study population was restricted to individuals who maintained the same INSTI and TAF combination before and during DOR use. Analyses described above were repeated, stratified by INSTI and TAF combination: (a) any INSTI with TAF, (b) any INSTI without TAF, (c) TAF without INSTI, (d) neither INSTI nor TAF. Only the first 36 months of follow-up were analyzed due to the insufficient number of data points beyond 36 months in this subset of the population.

## Results

### Study population

**Fig. 1:**
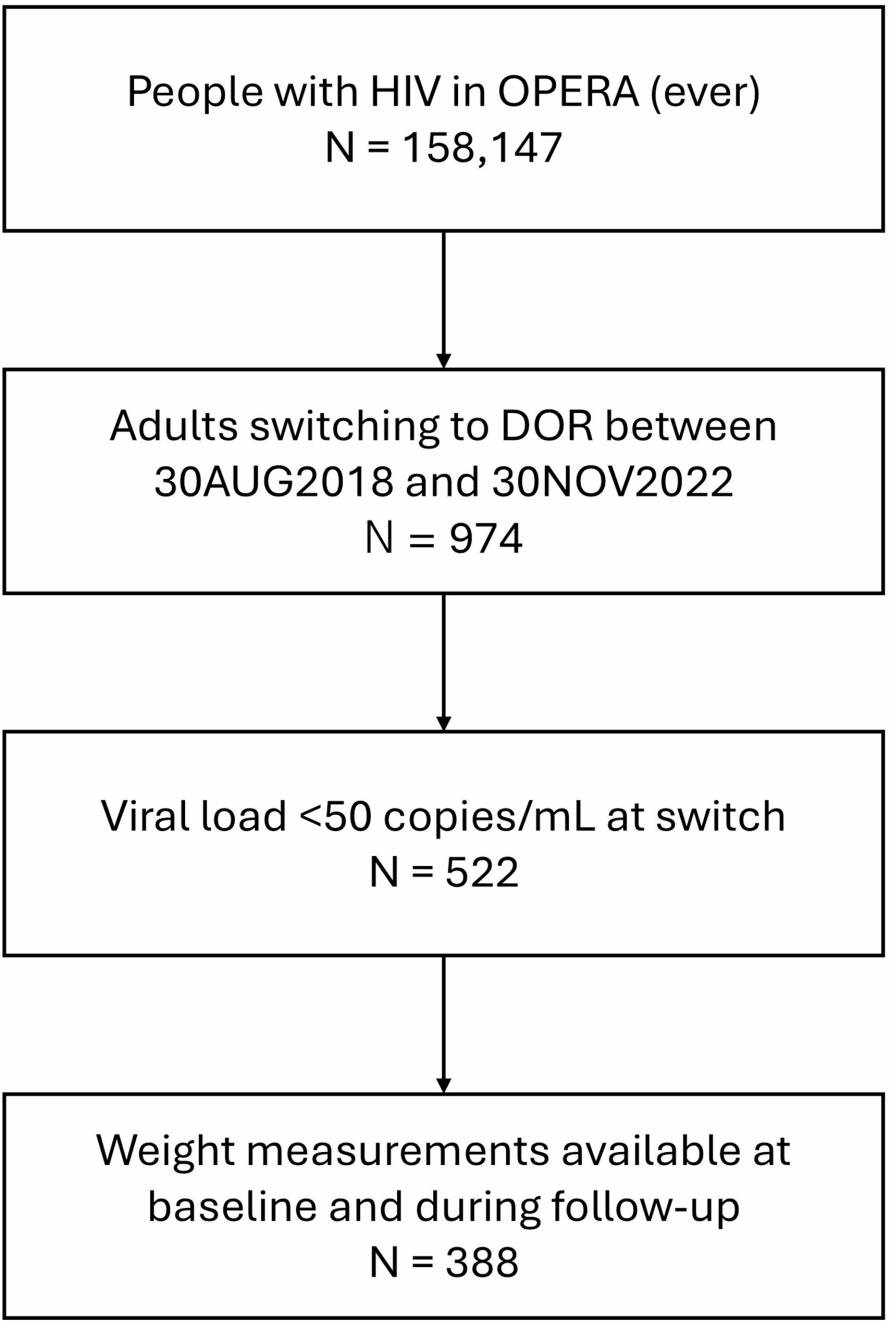
Inclusion in the study population DOR, doravirine; N, number

Of 974 ART-experienced adults with HIV in OPERA who switched to a DOR-containing regimen for the first time between 30AUG2018 and 30NOV2022, 388 met all inclusion criteria for the study (Fig. 1). Notably, 134 individuals (26% of those meeting all other inclusion criteria) were excluded due to a missing weight measurement either at baseline or during follow-up. The population was 21% women, 33% Black, and 24% Hispanic, and had a median age of 56 years at baseline (Table 1). Among women, 70% were Black, 12% were Hispanic, and 70% were of possible perimenopausal or menopausal age (≥ 40 years). Based on total cholesterol, 10% of the study population was experiencing dyslipidemia (≥ 240 mg/dL; Table 1). The median body weight at the time of switch was 85.7 kg; based on BMI, 39% of people in the study population were classified as overweight and 39% as obese (Table 1).

**Table 1:** Demographic and clinical characteristics at initiation of DOR-containing regimen

	Overall (*N* = 388)	Men (*N* = 305)	Women (*N* = 83)
Median age (IQR)	56 (44, 61)	56 (45, 61)	49 (38, 58)
Race, n (%)			
Black	128 (33)	70 (23)	58 (70)
White	231 (60)	209 (68)	22 (26)
Other/missing	29 (7)	26 (8)	≤ 5^†^
Hispanic ethnicity, n (%)	97 (25)	87 (28)	10 (12)
US Region, n (%)			
Northeast	42 (11)	35 (11)	7 (80
South	250 (64)	185 (61)	65 (78)
Midwest	19 (5)	16 (5)	≤ 5^†^
West	74 (19)	67 (22)	7 (8)
US Territories	≤ 5^†^	≤ 5^†^	≤ 5^†^
CD4 cell count (cells/µl) within 12 months of baseline			
Median (IQR)	621 (443, 858)	610 (412, 845)	657 (479, 964)
> 500, n (%)	252 (65)	194 (64)	58 (70)
≤ 500, n (%)	132 (34)	107 (35)	25 (30)
Missing, n (%)	≤ 5^†^	≤ 5^†^	0
Body weight (kg), median (IQR)	86 (75, 99)	85 (76, 97)	87 (73, 109)
BMI (kg/m^2^)			
Median (IQR)	28 (25, 33)	28 (25, 31)	34 (26, 39)
Underweight: <18.5, n (%)	6 (2)	≤ 5^†^	≤ 5^†^
Normal: ≥18.5 to < 25, n (%)	81 (21)	73 (24)	8 (10)
Overweight: ≥25 to < 30, n (%)	150 (39)	132 (43)	18 (22)
Obese: ≥30, n (%)	151 (39)	96 (31)	55 (66)
eGFR (mL/min/1.73m^2^)			
Median (IQR)	82 (60, 98)	82 (63, 97)	90 (59, 104)
≥ 90, n (%)	146 (38)	109 (36)	37 (45)
≥ 60 to < 90, n (%)	133 (34)	113 (37)	20 (24)
≥ 45 to < 60, n (%)	47 (12)	36 (12)	11 (13)
< 45, n (%)	40 (10)	31 (10)	9 (11)
Missing, n (%)	22 (6)	16 (5)	6 (7)
Total cholesterol (mg/dL)			
Median (IQR)	178 (148, 208)	176 (146, 204)	182 (158, 214)
Normal: <200, n (%)	240 (62)	194 (64)	46 (55)
Borderline Abnormal: ≥200 to < 240, n (%)	68 (18)	50 (16)	18 (22)
Dyslipidemia: ≥240, n (%)	38 (10)	28 (9)	10 (12)
Missing, n (%)	42 (11)	33 (11)	9 (11)
Diagnosis of diabetes mellitus (type 1 or 2), n (%)	91 (24)	74 (24)	17 (20)
Use of antihyperglycemics associated with weight loss,^‡^ n (%)	31 (8)	19 (6)	12 (14)
Use of antihyperglycemics associated with weight gain,^§^ n (%)	41 (11)	31 (10)	10 (12)
Invasive cancer, n (%)	67 (17)	59 (19)	8 (10)
History of AIDS defining events (ever), n (%)	175 (45)	149 (49)	26 (31)

µl, microliter; BMI, body mass index; DOR, doravirine; eGFR, estimated glomerular filtration rate; IQR, interquartile range; min, minute; N, number; US, United States

^†^ HIPAA regulations require the masking of cells with 1 to 5 individuals

^‡^ GLP-1 receptor agonist (albiglutide, dulaglutide, exenatide immediate or extended release, liraglutide, semaglutide subcutaneous or oral, tirzepatide, liraglutide/insulin degludec, lixisenatide, lixisenatide/insulin glargine), sodium-glucose co-transporter 2 inhibitors (bexagliflozin, canagliflozin, dapagliflozin, empagliflozin, ertugliflozin, canagliflozin/metformin, dapagliflozin/metformin, dapagliflozin/saxagliptin, empagliflozin/linagliptin, empagliflozin/metformin, empagliflozin/linagliptin/metformin, ertugliflozin/sitagliptin, ertugliflozin/metformin)

^§^ Insulin, chlorpropamide, gliclazide, glyburide, pioglitazone, rosiglitazone, repaglinide, tolbutamide, glimepiride, glipizide

### Characteristics of DOR-containing regimen and prior ART regimen

The single agent formulation of DOR, used in combination with other ARVs to form a complete regimen, was prescribed to 73% of the study population, while the remaining 27% of the population received the fixed dose combination of DOR/TDF/3TC. DOR was most commonly prescribed as part of complex ART regimens including several anchor agents (i.e., INSTI, protease inhibitor [PI], NNRTI, entry inhibitor): 47% were prescribed DOR plus ≥ 2 anchor agents and 23% were prescribed DOR plus one anchor agent (Table 2). Only 30% received DOR as part of a three-drug regimen with two nucleoside reverse transcriptase inhibitor (NRTI), of whom 92% received the fixed dose combination and 8% received the single agent. The NRTI backbone most frequently included TAF (38%) or TDF (29%).

Immediately prior to switching to DOR, 59% of individuals in the study population were using more than one anchor agent, whereas 25% were using an INSTI only, and 11% were using an NNRTI only (Table 2). Before the switch, the most common anchor agents used alone or in combination with other anchor agents were dolutegravir (39%), darunavir (34%), and bictegravir (24%). The NRTI backbone in the prior regimen included TAF for 60% of individuals (Table 2).

**Table 2:** Characteristics of ART regimens before and after switching to a DOR-containing regimen

	Overall (*N* = 388)	Men (*N* = 305)	Women (*N* = 83)
Anchor agent(s) prescribed immediately before switch to DOR, n (%)			
INSTI	98 (25)	75 (24)	23 (28)
PI	14 (4)	10 (3)	≤ 5^‡^
NNRTI	44 (11)	27 (9)	17 (20)
Other anchor agent or gap > 45 days	≤ 5^‡^	≤ 5^‡^	0
≥ 2 anchor agents	230 (59)	191 (63)	39 (47)
Anchor agent(s) prescribed with DOR, n (%)			
DOR alone (no other anchor agent)	117 (30)	76 (25)	41 (49)
DOR + INSTI	68 (18)	59 (19)	9 (11)
DOR + PI	10 (3)	10 (3)	0
DOR + NNRTI	9 (2)	7 (2)	≤ 5^‡^
DOR + Other^†^	≤ 5^‡^	0	≤ 5^‡^
DOR + ≥ 2 anchor agents	183 (47)	153 (50)	30 (36)
NRTI backbone agent(s) prescribed immediately before switch to DOR, n (%)			
TDF	37 (10)	34 (11)	≤ 5^‡^
TAF	233 (60)	173 (57)	60 (17)
Other	58 (15)	48 (16)	10 (12)
None or gap > 45 days	60 (15)	50 (83)	10 (12)
NRTI backbone agent(s) prescribed with DOR, n (%)			
TDF	113 (29)	80 (26)	33 (40)
TAF	148 (38)	116 (38)	32 (39)
Other	50 (13)	45 (15)	≤ 5^‡^
None	77 (20)	64 (21)	13 (16)
DOR formulation, n (%)			
Single agent	285 (73)	235 (77)	50 (60)
DOR/TDF/3TC fixed dose combination	103 (27)	70 (23)	33 (40)

3TC, lamivudine; ART, antiretroviral therapy; DOR, doravirine; INSTI, integrase strand transfer inhibitor; N, number; NRTI, nucleoside reverse transcriptase inhibitor; NNRTI, non-nucleoside reverse transcriptase inhibitor; PI, protease inhibitor; TAF, tenofovir alafenamide; TDF, tenofovir disoproxil fumarate

^†^ Entry inhibitor, capsid inhibitor

^‡^ HIPAA regulations require the masking of cells with 1 to 5 individuals

### Changes in body weight over follow-up

**Fig. 2:**
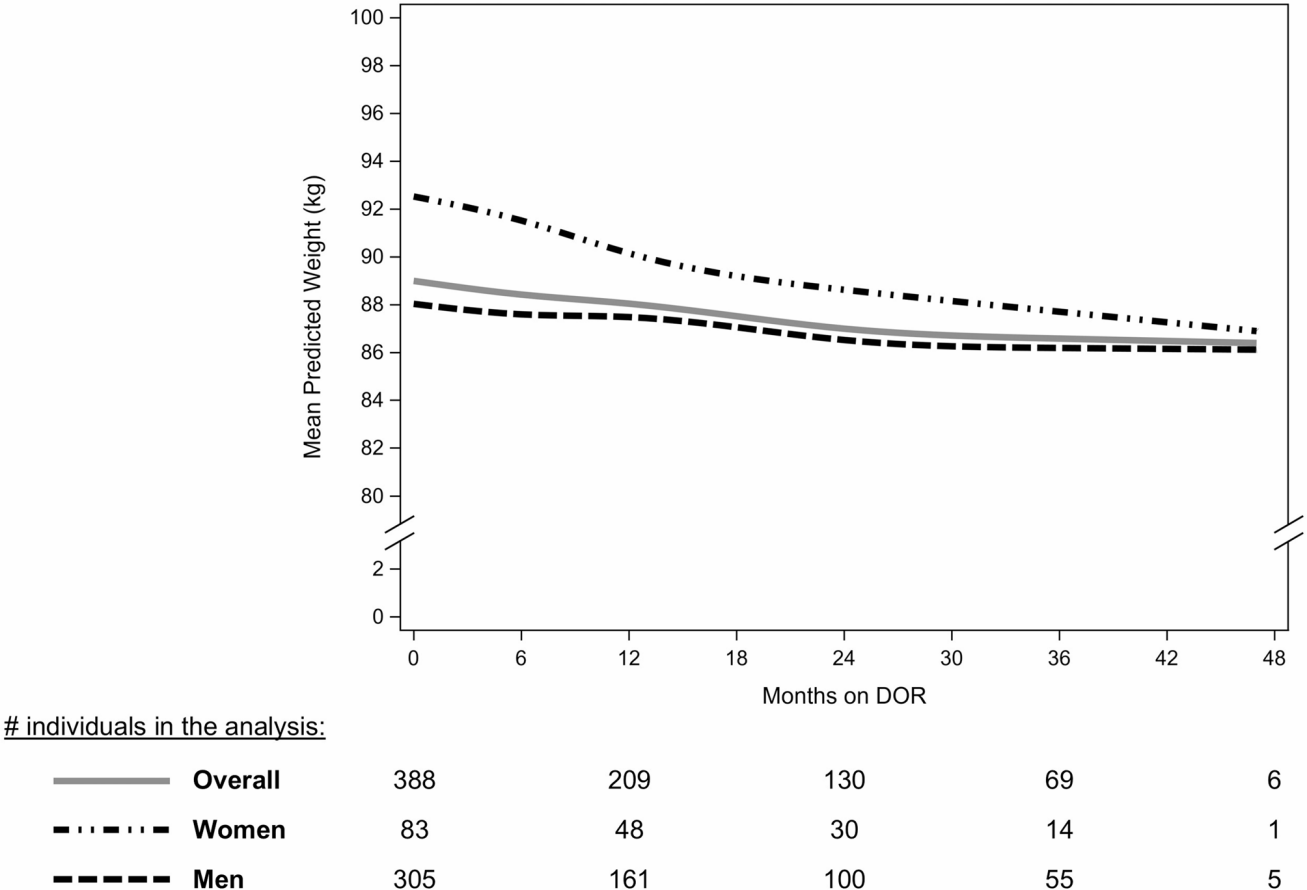
Predicted weight (kg) over months since switch to DOR, overall and stratified by sex. Mean predicted weights were estimated with a linear mixed model approach with restricted cubic splines on time (knots: 0, 6, 12, and 24 months) *#*, number; DOR, doravirine

The median follow-up time was 17.9 months (IQR: 8.0, 34.5). While 26% of the study population reached the end of the study with no change in regimen, 24% discontinued DOR and 47% changed other ARVs only. Weight loss was relatively constant throughout the entire study period (30AUG2018–31MAY2023; Fig. 2). In the overall study population, statistically significant weight loss of 0.80 kg/yr (95% CI: -1.32, -0.28) was observed. Both women and men experienced statistically significant and consistent weight loss over all follow-up (Fig. 2; Table 3). Women lost weight at a rate of -1.67 kg/year (95% CI: -3.32, -0.02), and men at a rate of -0.60 kg/year (95% CI: -1.12, -0.08; Fig. 2; Table 3).

**Table 3:** Estimated rate of change in weight over time since switch to DOR

	Total number of observations	Number of observations / person (IQR)	Rate of weight change, kg/year (95% CI)
Overall (*N* = 388)	2539	5 (2, 9)	-0.80 (-1.32, -0.28)
Stratification by sex			
Women (*N* = 83)	494	4 (2, 9)	-1.67 (-3.32, -0.02)
Men (*N* = 305)	2045	5 (2, 9)	-0.60 (-1.12, -0.08)
Stratification by TDF and/or EFV use before & after switch (*N* = 356)^†^			
TDF and/or EFV before & TDF after (*n* = 27)	452	7 (3, 11)	-1.66 (-4.07, 0.75)
No TDF or EFV before & after (*n* = 238)	1,539	5 (2, 9)	-0.72 (-1.31, -0.14)
TDF and/or EFV before only (*n* = 8)	50	4 (2, 10)	-0.71 (-2.40, 0.98)
TDF after only (*n* = 83)	498	5 (3, 8)	-2.22 (-4.03, -0.42)
Stratified by INSTI & TAF combination, if maintained (*N* = 232)^‡^			
INSTI with TAF (*n* = 100)	668	5 (2, 8)	-0.45 (-1.31, 0.41)
INSTI without TAF (*n* = 86)	540	6 (2, 8)	-0.72 (-1.50, 0.05)
TAF without INSTI (*n* = 28)	161	4 (2, 8)	-0.40 (-2.01, 1.21)
Neither INSTI nor TAF (*n* = 18)	107	5 (2, 8)	0.19 (-2.26, 2.65)

CI, confidence interval; DOR, doravirine; EFV, efavirenz; INSTI, integrase strand transfer inhibitor; IQR, interquartile range; N, number; TAF, tenofovir alafenamide; TDF, tenofovir disoproxil fumarate

^†^ Restricted to individuals with accurate information about EFV and TDF use

^‡^ Among people with HIV who maintained the same INSTI & TAF use combination before and after switching to DOR; restricted to the first 36 months of follow-up

### Sensitivity analyses

The first sensitivity analysis, stratified by EFV and/or TDF use, was restricted to 356 individuals, excluding 32 individuals with measurement error. The group of individuals who switched to TDF and DOR experienced the largest, statistically significant weight loss (-2.22 kg/yr; 95% CI: -4.03, -0.42). The group with neither TDF nor EFV before and after switch to DOR experienced a more modest, statistically significant weight loss (-0.72 kg/yr; 95% CI: -1.31, -0.14). No statistically significant changes were observed in the other groups, although trends towards weight loss were observed (Table 3).

The second sensitivity analysis, stratified by INSTI and TAF use combination, was restricted to 232 individuals who maintained the same INSTI and TAF use combination before and during DOR use. While there were no statistically significant changes in weight among any of the INSTI and TAF use combination groups, a trend towards weight loss was observed in all but the smaller group (neither INSTI nor TAF, *n* = 18; Table 3).

## Discussion

In one of the largest analyses of weight change after switching to a DOR-containing regimen in a real-world US setting and one of the first to focus on weight change by sex, moderate weight loss was observed in the overall population of 388 individuals. An average of 0.80 kg/year (95% CI: -1.32, -0.28) was lost while using DOR. After stratification, statistically significant weight loss was observed in both women and men. These findings provide important real-world evidence to inform clinical practice given that most individuals included were overweight (39%) or obese (39%) at switch to DOR, and that the expected yearly weight gain in the US population ranges from 0.5 kg to 1 kg per year [19].

Few prior studies have connected DOR use to weight loss as most randomized trials reported either no weight changes or only minor weight gain among individuals using DOR. In the DRIVE-SHIFT clinical trial of virologically suppressed adults who switched to once-daily DOR/3TC/TDF, slight weight gain consistent with the weight gain expected in people without HIV was observed although approximately 40% of individuals lost weight or had no change in weight through 144 weeks [20]. Other clinical trials of virologically suppressed individuals found no clinically or statistically significant weight changes after switch to a DOR-containing regimen [17, 20]. Early results from an ongoing double-blinded phase III trial investigating weight changes after switch to daily DOR/islatravir (ISL) from bictegravir/emtricitabine/tenofovir alafenamide have shown no statistically significant mean change in weight from baseline to week 48 attributable to DOR/ISL use [21]. In a similar study of switch to open-label DOR/ISL from various regimens, weight gain was observed on DOR/ISL through 48 weeks but was driven by removing weight-suppressive ART (i.e., EFV or TDF) in stratified analyses [22]. Taken together, the findings from randomized trials suggest that DOR may not have a significant effect on weight [23].

Due to the limited use of DOR since its recent approval, few real-world evidence studies have reached completion thus far. Like the present study, a recent study using retrospective chart review including 49 individuals from a tertiary care hospital in the US reported that weight change during the one-year follow-up period after switch to DOR was − 2.6% (95% CI: -5.1%, -0.1%) relative to pre-switch weight [24]. A multicenter study in Italy reported non-statistically significant weight loss through 24 weeks among 132 individuals switching to a DOR-containing regimen from any other prior regimen [25]. However, in a separate real-world study of 63 virologically suppressed people with HIV in Italy who switched to DOR/3TC/TDF from any prior ART regimen (mostly regimens containing an NNRTI [47%] or PI [32%] as the only anchor agent), a non-statistically significant median weight increase of 0.55 kg was observed over 12 months of follow-up [26]. A separate cohort study including 101 Black women living in South Africa found statistically significant weight gain through 48 weeks of follow-up after switching to DOR from EFV- or dolutegravir-based regimens [27, 28]. Given the inconsistent nature of these findings across real-world studies, there is still much to understand about the potential effect of DOR on weight in real-world settings.

In the present study, the most prominent weight loss was observed in women, with an average of 1.67 kg lost per year (95% CI: -3.32, -0.02). This weight loss was particularly noteworthy given the demographics of this group. Indeed, most women in the study population were likely perimenopausal or menopausal given the age distribution, and the majority were Black; both of which are groups that have been shown to disproportionately experience weight gain after initiation of ART. Indeed, several recent studies have highlighted the growing concern of weight gain associated with certain ART regimens [2, 5, 29], especially in women [6–8] and particularly in women of perimenopausal and menopausal age [9, 11, 30].

Sensitivity analyses were conducted in this study to assess the impact of other ARVs on DOR-associated weight loss. Specifically, weight gain has been associated with both INSTI and TAF use in both clinical trials and real-world observational studies [5, 31–34], while weight loss has been associated with use of TDF and EFV [2–4]. In this study, DOR remained statistically significantly associated with weight loss when EFV and TDF were absent both before and after switch, as well as when they were present in combination with DOR only. The point estimate for weight loss was largest when TDF was included after the switch only (-2.22 kg/yr). These findings suggest that while DOR appeared associated with a modest weight loss on its own, there may be an additive or synergistic association with TDF use. Non-statistically significant trends towards weight loss were also observed among a subset of the study population who maintained the same INSTI and TAF combination before and after switch. This suggests that at least some of the potential weight gain associated with INSTI and/or TAF use may have been offset by adding DOR.

This study is one of the first studies to examine the relationship between DOR use and weight change in a real-world US setting. To date, this study is among the largest published observational study on the topic with a total of 388 individuals, and among the first to focus on differences in weight change by sex. Stratification of analyses provided more granularity to interpret findings, and sensitivity analyses were conducted to account for the use of ARVs associated with either weight loss or weight gain to isolate the potential role of DOR. The study used data from the OPERA cohort, a large database of prospectively captured electronic health records from clinics across the US which includes a diverse and representative population of HIV care in the US. At the time of this study, OPERA included over 150,000 people with HIV, representing approximately 15% of people with HIV in the US and dependent areas.

While the overall sample size of 388 virologically undetectable individuals switching to DOR was large, stratification and restriction resulted in small sample sizes in some groups, reducing our ability to interpret changes in outcomes with confidence for those smaller groups. The lack of statistical significance in sensitivity analyses stratified by other ARV use may be attributed in part to the small sample sizes, especially in the groups who used TDF and/or EFV before switch only (*n* = 8), who used TDF and/or EFV before and TDF after switch (*n* = 27), who did not use any INSTI or TAF (*n* = 18), and who used TAF without any INSTIs (*n* = 28). Moreover, the duration of follow-up and availability of weight measurements beyond 36 months was limited. However, much of the weight change observed after switching ART regimens appears to occur within the first 48 weeks post-switch [2, 35]. Further, while the use of data from electronic health records provided prospectively collected data reflecting real-world clinical practices, it was limited by lack of data on reasons for regimen changes and potential measurement error. Indeed, some providers did not always enter a stop date in the EHR when a regimen was modified, which can result in misclassification of regimens. This limitation was addressed by excluding individuals on improbable regimens from the relevant analyses.

This study was descriptive in nature and did not include a comparison group for clinical outcomes over time. Because of this, no inference could be drawn regarding weight changes after switch to DOR relative to other regimens. Moreover, statistical analyses were not adjusted for potential confounding other than stratification by sex, TDF and/or EFV use, and INSTI and TAF use combination, as well as restriction to individuals maintaining the same combination of INSTI and TAF use before and after switch. Perimenopause and menopause were not measured directly, but rather used age as an imperfect proxy. Analyses were not adjusted to control for women’s menopausal status, which is associated with weight gain. Lifestyle factors such as diet and exercise were not available and therefore could not be described or included in the statistical models. However, the study aimed to present average changes in weight in a population that is representative of people with HIV in care in the US. The inclusion of people with diverse individual characteristics may be helpful to clinicians as it reflects real-world clinical practice. Virologic outcomes during DOR use were not assessed for this study. While there is evidence that higher viral loads at baseline are associated with higher weight gain, it is unlikely that loss of virologic control in this population of virologically suppressed individuals switching to DOR would explain the changes in weight observed over a median of 17.9 months [2].

Of the 522 virologically suppressed adults who switched to DOR during the study period and were considered for inclusion in the study, 134 (26%) were excluded due to a missing weight measurement either at baseline or during follow-up. It is possible that weight monitoring was a lower priority for individuals with normal weight, as suggested by the fact that nearly 80% of the population was overweight or obese at the time of switch to DOR and that a large proportion of DOR users were excluded due to the absence of weight measurement. The resulting lack of weight measurements could have led to selection bias as several studies have demonstrated that overweight and obesity are associated with decreased risk of additional weight gain [36–38]. However, while weight is usually measured at each in-person visit in the US, regardless of a person’s weight, it may not be available for virtual consultations. The high proportion of individuals who were overweight or obese may also be due, in part, to the funneling of overweight and obese individuals towards DOR and away from INSTI- and TAF-containing regimens, for which associations with weight gain have been widely discussed [2–4].

Of note, 70% of individuals in the study were taking DOR in addition to another anchor agent, suggesting that in the US, DOR is mainly prescribed to highly treatment experienced individuals necessitating a complex ART regimen [39, 40]. While the combination of DOR with other anchor agents complicates the interpretation of findings, sensitivity analyses controlling for the use of agents associated with weight gain or weight loss were conducted to alleviate this concern.

## Conclusions

This is one of the first real-world analyses that reveals a clinically and statistically significant weight loss among virologically suppressed individuals who switched to a DOR-containing regimen in the US. A high proportion of individuals were on complex regimens and were overweight or obese. Importantly, pronounced weight loss was observed among women. Patterns of use of ARVs associated with weight gain (INSTI and/or TAF) or weight loss (TDF and/or EFV) did not fully explain the weight loss observed on DOR. The modest weight loss observed after switching to DOR is clinically meaningful given that most individuals included were overweight or obese at switch to DOR and that weight gain has often been associated with female sex, especially during perimenopause and menopause. A switch to DOR may thus be an interesting treatment option where weight gain is a concern.

## Data Availability

The datasets used in this study are not publicly available due to privacy concerns and the proprietary nature of the database but can be accessed upon reasonable request through the corresponding author to the OPERA Epidemiological and Clinical Advisory Board. Access to codes may be granted upon request with parties agreeing to privacy restrictions and technological specifications and requirements.

## Declarations

### Abbreviations

µl Microliter.

3TC Lamivudine.

AIDS Acquired immunodeficiency syndrome.

ARV Antiretroviral.

BMI Body mass index.

CI Confidence interval.

dL Deciliter.

DOR Doravirine.

EFV Efavirenz.

eGFR Estimated glomerular filtration rate.

FDA Food and Drug Administration.

HIPAA Health Insurance Portability and Accountability Act.

HITECH Health Information Technology for Economic and Clinical Health.

HIV Human immunodeficiency virus.

INSTI Integrase strand transfer inhibitor.

IQR Interquartile range.

IRB Institutional Review Board.

kg Kilogram.

N Number.

NNRTI Non-nucleoside reverse transcriptase inhibitor.

NRTI Nucleoside reverse transcriptase inhibitor.

OPERA Observational Pharmaco-Epidemiology Research and Analysis.

PI Protease inhibitor.

TAF Tenofovir alafenamide.

TDF Tenofovir disoproxil fumarate.

US United States.
